# Supplementary material for: Deep Learning-Based Segmentation of Head and Neck Organs-at-Risk with Clinical Partially Labeled Data
Source: Entropy (Basel). 2022 Nov 15;24(11):1661. doi: 10.3390/e24111661 (PMC9689629; doi:10.3390/e24111661)
Supplement: Supplementary file 1 [file entropy-24-01661-s001.zip › entropy-1974415-supplementary.pdf]

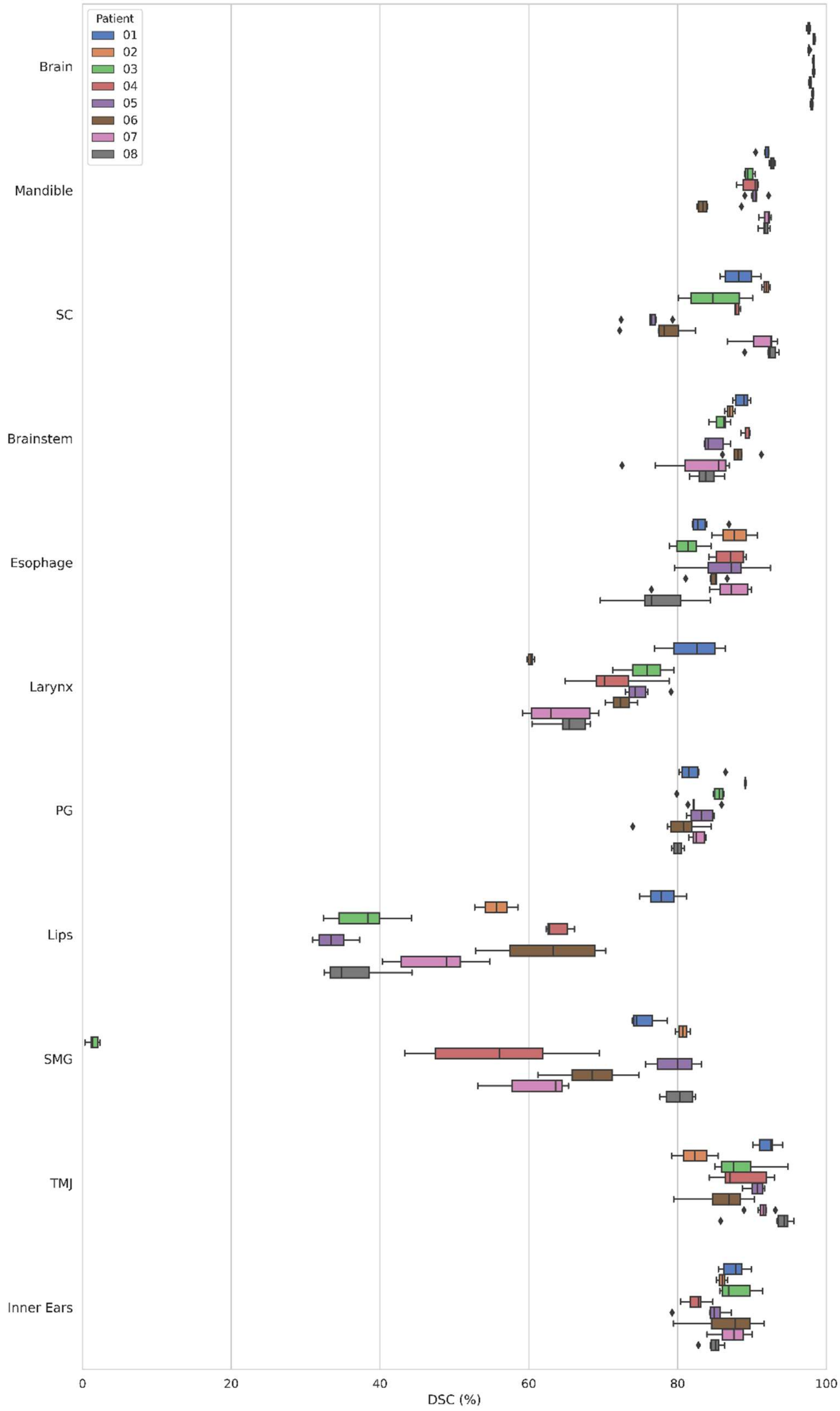

Figure S1. Analysis of the DSC per OAR and patient obtained with the trained semi-supervised nnU-Net.

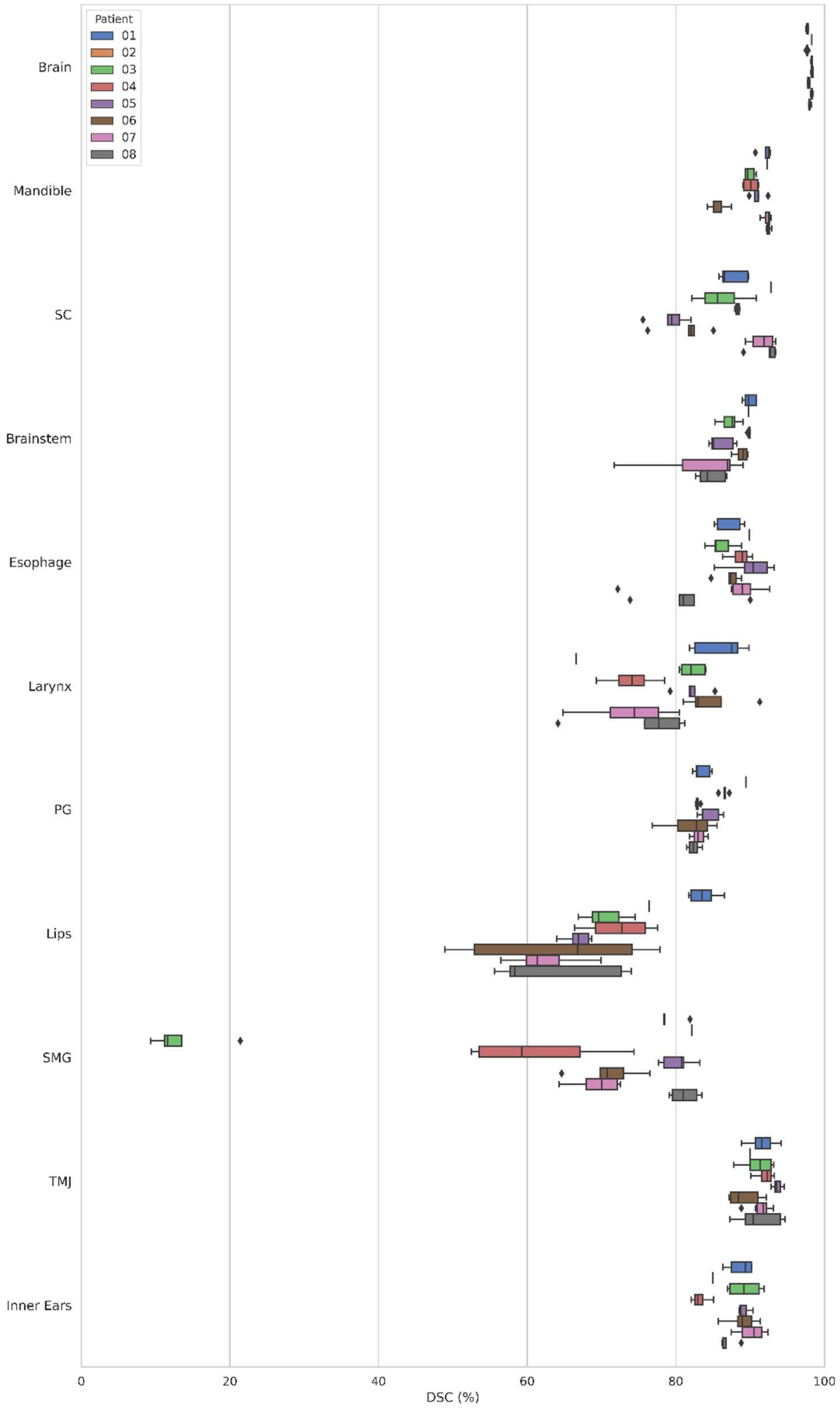

Figure S2. Analysis of the DSC per OAR and patient obtained with the trained self-supervised nnU-Net (including a baseline CT image from each patient in the training).
